# IL-22 is rapidly induced by Pathogen Recognition Receptors Stimulation in Bone-Marrow-derived Dendritic Cells in the Absence of IL-23

**DOI:** 10.1038/srep33900

**Published:** 2016-09-22

**Authors:** Silvia Fumagalli, Anna Torri, Angela Papagna, Stefania Citterio, Federica Mainoldi, Maria Foti

**Affiliations:** 1 School of Medicine and Surgery, University of Milano-Bicocca, Milan, 20126, Italy; 2 Department of Biotechnology and Bioscience, University of Milano-Bicocca, Milan, 20126, Italy

## Abstract

In vertebrates, microorganisms are recognized by pathogen recognition receptors (PRRs). Exposure of immune cells to the ligands of these receptors activates intracellular signaling cascades that rapidly induce the expression of a variety of genes. Within these genes, the cytokines family plays a crucial function because of its role in adaptive immunity induction and in tissue-specific functional regulation, such as tissue repair and tissue homeostasis during steady state conditions. Within the myeloid compartment, dendritic cells (DCs) release a variety of inflammatory cytokines in response to microbes. In this study, we show that BMDCs release IL-22 directly upon PRRs activation without the need of IL-23 signaling as reported for other IL22-producing cells. Moreover, we demonstrate that cytokine IL-22 is rapidly released in a cell-specific manner as macrophages are not able to produce IL-22 through the same PRRs system. In addition, we characterize the intracellular signaling cascade required for IL-22 release in BMDCs. Myd88, MEK1/2, NFkb and AhR, but not p38, NFAT, and RORgt, were found to be involved in IL-22 regulation in DCs. Our study suggests that BMDCs possess a unique intracellular molecular plasticity which, once activated, directs different BMDCs functions in a cell-specific manner.

Pathogen recognition receptors (PRRs) stimulation induces the release of critical pro-inflammatory cytokines necessary to activate potent immune responses. The induction of specific cytokines by cells of the innate immunity early during infection influences the magnitude of the nascent inflammatory response and directs the type of adaptive response that provides best sterilizing immunity. Therefore, understanding the molecular intracellular pathways to PRRs stimulation will provide information about the basic mechanisms of how immunity is developed. Moreover, it will also provide an understanding of target-specific pathways and help to control the inflammatory process itself and the nascent adaptive response required.

Immunity to microbes has provided an extensive understanding of the complexity operating within the immune system. Microbial insults induce both common and specific activatory pathways in cells of the immune system as a result of a steady adaptation of the mutual interaction between microbes and their host.

The intracellular pathways induced by PRRs have been intensively studied. The downstream transcription factors induced, such as NF-KB, AP-1, IRFs, and MAPKs signaling pathways, play a major role in cytokine gene expression in a number of cellular systems. However, the precise mechanism governing cytokine gene regulation in a cell-specific context following PRR activation is still poorly understood. Dendritic Cells (DCs) reside in the peripheral tissues and the secondary lymphoid organs in an immature state and act as a “sensors” of a number of “danger” and/or “self/non self” signals. Particular interest has been directed towards the molecular profiles between dendritic Cells (DCs) and live bacteria^1^,^2^,^3^,^4^. Specific receptors, signaling pathways and response types under specific microbial interactions are currently being studied intensively. DCs are located at the anatomical interface where first microbial contact occurs and are therefore supposed to interact with microbial pathogens in the early stages. They also actively participate in the regulation of the inflammatory process together with lymphoid and myeloid cells and in the induction of the most appropriate adaptive response^5^,^6^.

Analysis of genes induced in DCs in response to microbes is used to predict the impact of such interactions on DCs activation^1^,^7^. Our study shows that a common genetic program is induced in DCs by live Gram+ and Gram- bacteria, including the induction of cytokines of the IL10 family such as IL-22. IL-22 is a recently discovered cytokine which regulates innate responses at the epithelial barriers interface. Immune cells from both the innate and the adaptive immunity are able to release the cytokine IL-22, although with different efficiency^8^,^9^. IL-22 expression is restricted to immune cell lineages, whereas the functional IL22R receptor seems to be restricted to the stromal cell compartments of different tissues and organs, such as skin, pancreas, intestines, liver, lung, and kidney^10^,^11^. IL-22 is important for the induction of genes involved in tissue inflammation, immunosurveillance, and homeostasis^11^,^12^,^13^,^14^,^15^,^16^,^17^,^18^,^19^,^20^.

In mice, IL22 is both expressed by innate and adaptive immune system cells. The innate cellular source of the cytokine includes NK cells, NKT cells, γδ T cells, innate lymphoid cells (ILCs), and CD11c+ DCs^14^,^16^,^21^,^22^,^23^. Induction of IL-22 is mainly initiated by IL-23 released by antigen-presenting cells (APC) following PRR stimulation^24^,^25^,^26^.

In DCs, cytokines are produced following the binding of PRRs or endogenous danger signals to TLRs, NOD-like receptors, and C-type lectin receptors, such as dectin-1^27^,^28^. However, the induction of specific cytokines (e.g. IL-12 or IL-23) is regulated by TLR and C-type lectin receptors in DCs^29^. NFkB, IFN regulatory factor (IRF)-1, and IRF-8 signaling pathways play important roles in the expression of IL-12^29^,^30^,^31^,^32^. In contrast, induction of IL6- and IL-23 in DCs by dectin-1 agonist is mediated by signaling via Syk and CARD9^33^.

In this study, we show that BMDCs directly release IL-22 upon PRRs activation without the need of IL-23 signaling, in contrast to previously described cellular systems (T Cell, NK cells, LTi cells). We further identify signaling requirements and mechanisms of IL-22 release in BMDCs. We examine the role of a number of signaling molecules downstream to PRR activation and show that the absence of Myd88 or inhibition of ERK-MEK1/JNK suppresses IL-22 production by BMDCs stimulation with TLRs and dectin-1 agonists. Similar patterns of cytokine regulation by the Myd88 and ERK-MEK/JNK signaling pathways are observed in splenic CD11c+ cells isolated from wild type mice. In contrast, IL-23 production was not affected by inhibition of ERK-MEK1/JNK activation. We conclude that multiple intracellular signaling pathways not operating on macrophages control IL-22 cytokine release in BMDCs without the requirement of IL-23, underlining a direct role of DCs in the initiation of inflammatory as well as in the tissue remodelling programs in response to infection-induced signals.

These findings provide new evidence that IL-22 and IL-23 production use distinct signaling pathways in DCs. We therefore suggest ERK-MEK1/JNK signaling as an important target for new therapeutic interventions to control IL-22/IL-22R axes in the treatment of persistent infections, chronic inflammation, and autoimmune diseases.

## Results

### IL-22 is differentially regulated by whole live bacteria in dendritic cells

Interactions between dendritic cells (DCs) and microbial pathogens are fundamental to the generation of innate and adaptive immune responses. To define the molecular events underlining host-bacteria interactions, we initiated a whole-genome investigation of DCs response to live bacteria. D1 cells^34^ have been activated with live *Listeria monocytogenes, Listeria innocua, Lactobacillus paracasei, and Lactococcus lactis* at the multiplicity of infection (MOI) that induced the optimal DCs activation as assessed by FACS analysis (data not shown). Surprisingly, we detected the modulation of the IL-22 gene by most of the bacteria analyzed.

**Figure 1:**
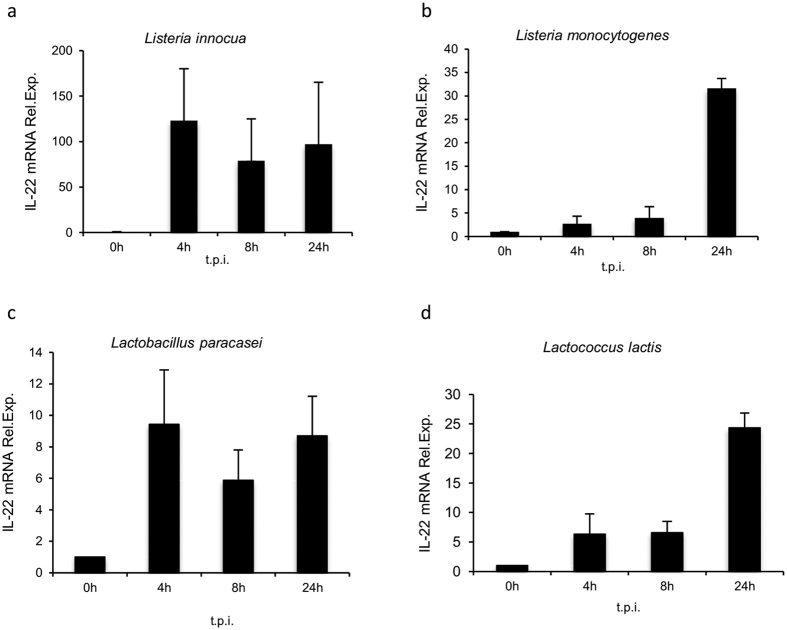
IL-22 mRNA expression is induced in D1 cells infected with bacteria. D1 cells (4,5 × 10^5^ cells/ml) were infected with (**a**) *L. innocua* (MOI 1:1000); (**b**) *L. monocytogenes* (MOI 1:20); (**c**) *L. paracasei* (MOI 1:1000); (**d**) *L. lactis* (1:1000). IL-22 mRNA was assessed by qRT-PCR at 4 h, 8 h and 24 h post infection. Target mRNA was normalized to 18S and expressed as fold stimulation over control (0 h, non- treated cells). t.p.i.: time post infection. Each measurement was performed in duplicate. The data represent the mean values of two independent experiments (±SD) for (**a**,**b**) and three experiments (±SD) for (**c**,**d**).

To understand cytokine regulation, D1 cells were treated with bacteria at different time post infection (t.p.i.) and its expression was studied by real-time RT-PCR analysis. Although IL-22 was induced by all tested bacteria, only *Listeria innocua* and *Lactobacillus paracasei* were able to sustain IL-22 gene expression in DCs from 4 h to 24 h t.p.i. (Fig. 1a,c). In contrast, *Listeria monocytogenes* and *Lactococcus lactis* induced maximal gene expression at 24 h post infection, suggesting that DCs are able to differentially respond to different gram-positive bacteria, specifically with a different cytokine production kinetics (Fig. 1b–d and Supplementary Fig. 1a,b).

**Figure 2:**
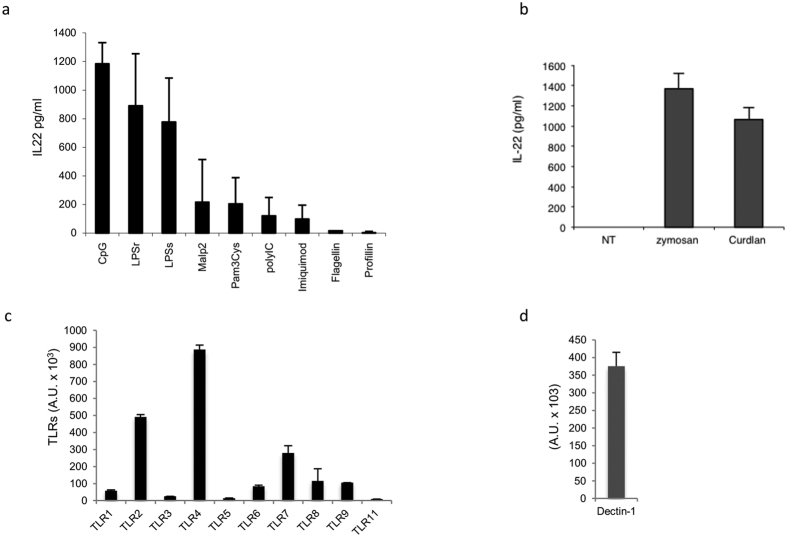
PRRs activation leads to IL-22 production in BMDCs. (**a**) BMDCs (1,5 × 10^6^ cells/ml) were stimulated with different TLR agonists: CpG (5 ug/ml), LPSr (5 ug/ml), LPSs (5 ug/ml), MALP2 (100 ng/ml), Pam3Cys (100 ng/ml), polyIC (1 ug/ml), imiquimod (1 ug/ml), flagellin (50 ng/ml), and profillin (100 ng/ml). (**b**) BMDCs were stimulated with zymosan (10 ug/ml) and curdlan (10 ug/ml). After 20 h, supernatants were collected and tested for IL-22 production by ELISA. TLRs (**c**) and Dectin-1 (**d**) expression in BMDCs. At day 8 of culture, BMDC cells were collected in trizol and qRT-PCR was performed. Each measurement was performed in duplicate. Samples were normalized with 18S expression levels and expressed as arbitrary units. The data represent mean values of three independent experiments (±SD).

Induction of IL-22 by bacteria in DCs prompted us to investigate the type of receptor molecule involved in this particular cytokine induction. Bone-marrow derived dendritic cells (BMDCs) were stimulated with synthetic ligands to activate Toll-like receptors (TLRs). Different TLR agonists were tested for their ability to induce IL-22 in BMDCs. CpG (TLR9), smooth LPS (sLPS), and rough LPS (rLPS) for TLR4 stimulation; poly:IC (TLR3), Pam3Cys (TLR1/TLR2), MALP2 (TLR2/TLR6), imiquimod (TLR7), Profillin (TLR11), and flagellin (TLR5) were used to activate the cells. After 20 h of incubation, IL-22 secretion was measured by ELISA. TLR4 and TLR9 agonists showed the highest ability to induce IL-22 in BMDCs compared to TLR2/TLR1, TLR2/TLR6 dimers and TLR3 agonists (Fig. 2a and Supplementary Fig. 1c,d). PRR agonists activation was characterized by a dose-response experiment, further confirming a positive correlation between the amount of IL-22 production and TLR agonists concentration (Supplementary Figs 2 and 3). TLR2 ligation by MALP2 or Pam3Cys did not play a major role in IL-22 induction as only a moderate production of the cytokine in BMDCs was measured upon stimulation (Fig. 2a).

In contrast, TLR5 and TLR11 ligands did not induce any cytokine secretion, indicating that these receptors were either not involved in IL-22 production or not optimally expressed in BMDCs. Indeed, TLR5 and TLR11 receptors expression analysis revealed that these receptors were not amplified in BMDCs (Fig. 2c).

To explore the possibility that other pattern recognition receptors (PRRs) are able to trigger IL-22 in BMDCs, we examined the effect of ligands that simulate the response to fungi. The addition of zymosan (TLR2/Dectin-1) and curdlan, a specific dectin-1 agonist, induced the release of IL-22 into BMDCs supernatants within 20 h of stimulation (Fig. 2b). Because of a low IL-22 induction by TLR2 ligands (Fig. 2a) and the consistently high induction by curdlan, we concluded that the contribution of dectin-1 was responsible for the IL-22 release in BMDCs, although TLR2 and dectin-1 receptors expression in this cell type was actually comparable (Fig. 2c,d).

### PRR-mediated IL-22 secretion is cell-type specific and does not require IL-23 in BMDC

**Figure 3:**
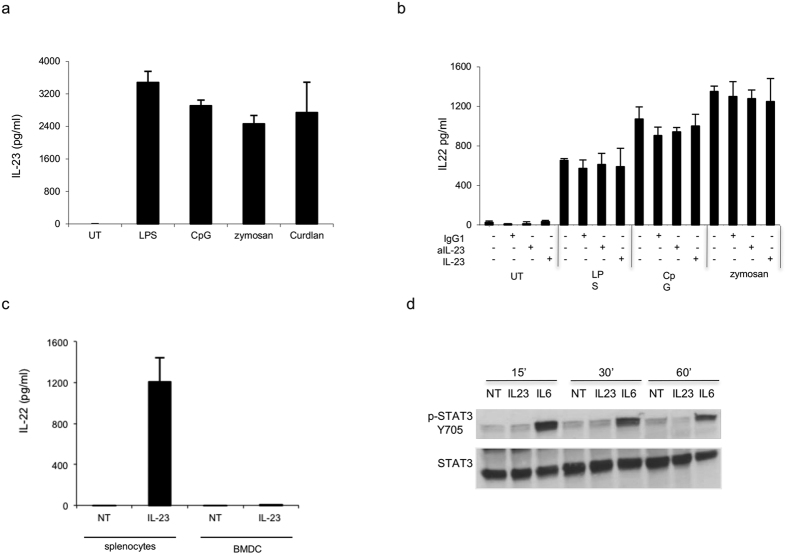
IL-22 production in BMDCs is IL-23-independent. (**a**) BMDCs (1,5 × 10^6^ cells/ml) were stimulated with LPS (5 ug/ml), CpG (5 ug/ml), zymosan (10 ug/ml), and curdlan (10 ug/ml). After 20 h, supernatants were collected and tested for IL-23 production by ELISA. (**b**) BMDC were stimulated in presence of LPS (5 ug/ml), CpG (5 ug/ml), and zymosan (10 ug/ml) in the presence or absence of an antibody against IL-23 (2 ug/ml) (aIL-23), IgG1 (2 ug/ml), and rmIL-23 (5 ng/ml). After 20 h, IL-22 production was tested by ELISA. (**c**) BMDCs (1,5 × 10^6^ cells/ml) and splenocytes (2 × 10^6^ cells/ml) were stimulated with rmIL-23 (20 ng/ml). After 20 h, supernatants were collected and tested for IL-22 production by ELISA assay. (**d**) Stat 3 phosphorylation of BMDCs treated with rmIL-23 (20 ng/ml) or rmIL-6 (20 ng/ml) for the indicated time point. BMDC extracts were prepared and analyzed by Western Blot analysis. The data represent mean values of three independent measurements (±SD).

Several studies have demonstrated that IL-23 induces IL-22 in innate lymphoid and in Th17 cells *in vivo*^14^,^35^. Therefore, to determine whether IL-22 was mediated directly by PRRs stimulation or indirectly induced by IL-23 released following receptor stimulation, we determined the amount of IL-23 in response to LPS, CpG, and zymosan. As expected, IL-23 was induced by TLRs and C-type lectin ligands tested (Fig. 3a), suggesting that IL-22 release may be mediated by IL-23. The production of IL-22 induced by LPS, CpG, and zymosan in the presence or absence of a neutralizing antibody to IL-23 after 20 h in BMDCs was measured. We were not able to detect any reduction of IL-22 in the presence of the anti-IL-23 antibody (Fig. 3b). In addition, we incubated BMDCs directly with recombinant IL-23 (rIL-23) for 20 h and subsequently measured IL-22 by ELISA. BMDCs were unable to release IL-22 in response to rIL-23 in contrast to splenic cells used as positive control (Fig. 3c). Moreover, treatment with rIL-23 did not result in STAT3 phosphorylation (Fig. 3d), leading to the conclusion that BMDCs are not responsive to IL-23 in these experimental settings although the IL23R complex was expressed (Supplementary Fig. 4).

**Figure 4:**
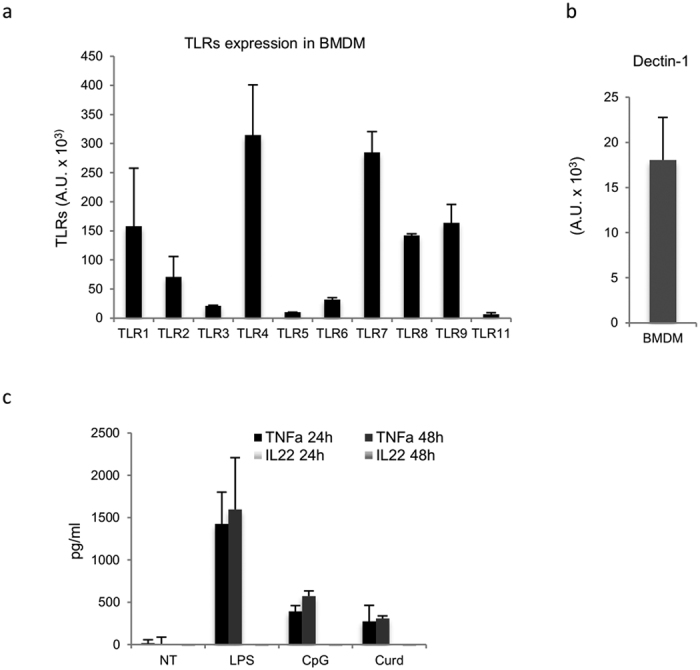
PRRs activation does not induce IL-22 in BMDM. TLRs (**a**) and dectin-1 (**b**) expression in BMDM. At day 8 of culture, cells were collected in trizol and qRT-PCR was performed. Each measurement was performed in duplicate. Samples were normalized with 18S expression levels and expressed as arbitrary units. (**c**) BMDM cells (1,5 × 10^6^ cells/ml) were stimulated with LPS (5 ug/ml), CpG (5 ug/ml), and curdlan (10 ug/ml). After 24 h and 48 h, supernatants were collected and tested for TNFa and IL-22 production by ELISA. The Data represent the mean values of three independent measurements for panels (**a**,**b**), and two independent experiments for (**c**).

The existence of a specific PRR-signaling inducing IL-22 in BMDCs prompted us to investigate whether cell types of the innate immunity other than BMDCs are able to release IL-22 in response to PRRs stimulation. For this reason, we generated bone marrow-derived macrophages (BMDMs) by culturing cells in medium containing M-CSF^36^. First, we verified that PRR receptors were expressed by BMDMs via qRT-PCR analysis (Fig. 4a,b) and then tested IL-22 induction. Cells were treated with LPS, CpG, and curdlan after 24 h and 48 h of stimulation. Surprisingly, we did not detect any IL-22 release from BMDMs; however, the cells were able to release TNFα after PRR receptors stimulation (Fig. 4c). We corroborated these results by measuring IL-22 release in *ex vivo* macrophage populations derived from either the peritoneum (F4/80+ cells) or the spleen (CD11b+ cells) upon PRR receptors activation (data not shown). The absence of IL-22 detection in all these cellular systems suggests that a unique PRR-induced IL-22 pathway is operating exclusively in BMDCs.

Therefore, it is tempting to speculate that BMDCs possess a unique capability to produce IL-22 in response to PRR receptors stimulation. The precise differences between cell surface receptors and/or intracellular signaling molecules operating in BMDCs compared to macrophages following PRR activation are presently under investigation.

### TLR-induced secretion of IL-22 requires Myd88 signaling

**Figure 5:**
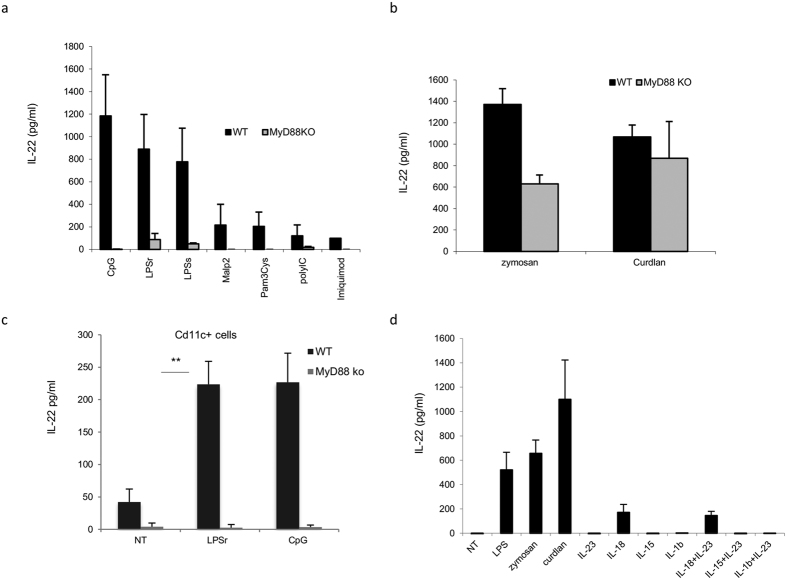
IL-22 production in BMDCs and splenic CD11c+ cells is MyD88 dependent. (**a**) BMDCs (1,5 × 10^6^ cells/ml) from both WT and MyD88 KO mice were stimulated with CpG (5 ug/ml), LPS (5 ug/ml), Malp2 (5 ug/ml), Pam3Cys (5 ug/ml), PolyI:C (5 ug/ml), imiquimod (1 ug/ml) and (**b**) with zymosan (10 ug/ml) and curdlan (10 ug/ml). (**c**) CD11c+ cells were purified from the spleens of a group of mice (N = 3). CD11c+ cells were then stimulated with LPSr (5ug/ml), CpG (5ug/ml). After 20 h, supernatants were collected and tested for IL-22 production by ELISA (**d**) BMDCs were stimulated with the cytokines IL-23, IL-18, IL-1b, IL-15 and their combinations. After 20 h, supernatants were collected and tested by ELISA. The data (**a**,**b**) represent mean values of four independent measurements (±SD); data in (**c**) represent mean values of three independent measurements (±SD); data in (**d**) represent mean values of two independent measurements (±SD). Student’s T test statistical significance is shown (**p < 0.01).

Myd88 plays a crucial role in TLRs and IL-1R signaling pathways. Therefore, we evaluated the impact of this adaptor molecule on IL-22 secretion. Cytokine production in Myd88−/− mice was determined. BMDCs were stimulated with LPS, CpG, zymosan, and curdlan, and the protein level was measured by ELISA. A strong difference in the level of IL-22 between WT and Myd88−/− animals during TLR receptor stimulation was revealed (Fig. 5a). As expected, this difference was absent when cells were stimulated with curdlan (C-type receptors), confirming a role of Myd88 in the regulation of the expression of IL-22 following TLRs, but not dectin-1 stimulation (Fig. 5b). To verify that Myd88 is also required by *ex vivo* DCs, we stimulated splenic CD11c+ cells with LPS and CpG from wt and Myd88−/− mice. Again, a strong reduction of IL22 in Myd88−/− animals was observed, confirming the need for the Myd88 adaptor for cytokine production also in *ex vivo* cells (Fig. 5c).

Because Myd88 may control also IL-1R or IL-18R signaling, we treated the cells with rIL-1b and rIL-18 for 24 h and measured cytokine secretion in BMDCs. We were able to measure 200 pg/ml of IL-22 only under IL-18 stimulation (Fig. 5d). This suggests that IL-22 release is partly controlled by IL-18. Nevertheless, the overall IL-22 production was not totally replaced by IL-18, indicating that additional factors are required for cytokine secretion during PRR stimulation in BMDCs.

### MAP Kinases ERK, JNK, and p38 differentially contribute to PRRs driven IL-22 secretion in BMDCs after stimulation

Production of cytokines is dependent on the activation of MAPK pathways for the induction of the transcription factors AP-1 upon PRRs stimulation.

**Figure 6:**
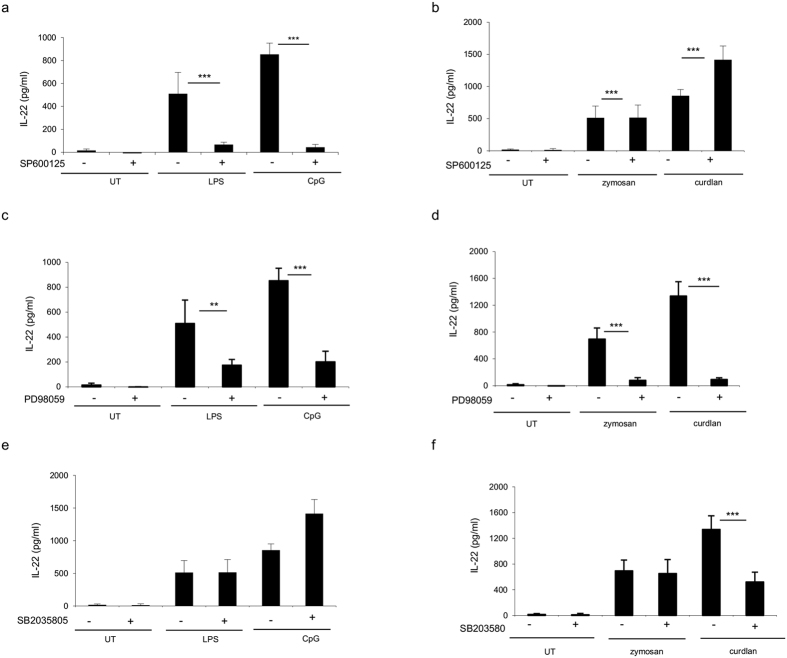
JNK and ERK, but not p38 MAP kinase, are involved in IL-22 production in BMDCs. BMDCs (1,5 × 10^6^cells/ml) were pretreated for 30 min with (**a**,**b**) JNK inhibitor SP600125 (20 uM), (**c**,**d**) ERK inhibitor PD98059 (20 uM), and (E,F) p38 inhibitor SB203580 (20 uM). BMDCs were then stimulated with LPS (5 ug/ml), CpG (5 ug/ml), zymosan (10 ug/ml), and curdlan (10 ug/ml). After 20 h, supernatants were collected and tested for IL-22 production by ELISA. The data represent mean values of three independent measurements (±SD). Student’s T test statistical significance is shown (***p < 0.001; **p < 0.01; *p < 0.05).

Therefore, we examined the signaling pathways used by TLRs and dectin-1 ligands to promote IL-22 production in BMDCs. BMDCs were pretreated for 30 min with different inhibitors, such as SP600125 (targeting c-Jun N-terminal kinases), PD98059 (MEK1–ERK inhibitor), and SB203580 (targeting p38 MAPK), and subsequently cultured with LPS (5 μg/ml), CpG (5 μg/ml) or zymosan and curdlan (10 μg/ml). The production of IL-22 was determined by ELISA after 20 hr (Fig. 6). The inhibitor concentrations used did not show any relevant toxicity to the cells (data not shown). The JNK and ERK/MEK1 inhibitors suppressed PRR ligands-induced IL-22 expression in BMDCs (Fig. 6a–d). However, the p38 inhibitor had no significant effect on the induction of IL-22 after stimulation with LPS, CpG, and zymosan, whereas a reduction was observed when cells were stimulated with curdlan (Fig. 6f). Therefore, the expression of IL-22, mediated by LPS, CpG, zymosan and curdlan, is controlled by both JNK and MEK1-ERK kinases. In contrast, p38 controls IL-22 expression induced by curdlan, but not by LPS, CpG, and zymosan (Fig. 6e,f).

The results clearly indicate that AP-1 induction mediated by JNK, ERK/MEK1, but not by p38, is essential to obtain IL-22 gene transcription triggered by PRR ligands. In addition, p38 seams to participate in IL-22 regulation only during dectin-1 stimulation. To test specificity in the intracellular signaling induction, the involvement of the MAPK pathways during induction of IL-23 was further determined. In contrast to IL-22, the suppressive effect of JNK inhibitor on IL-23 was observed only during zymosan and curdlan, but not during LPS and CpG stimulation (Supplementary Fig. 5a,b). Similarly, inhibition of ERK/MEK1 did not affect IL-23 induction in the presence of any of the PRR ligands as it was observed for IL-22 (Supplementary Fig. 5c,d). P38 suppression affected IL-23 in response to curdlan as it was observed for IL-22; nevertheless, p38 was required for IL-23 induction as opposed to IL-22 when cells were stimulated with LPS (Supplementary Fig. 5e). PRR agonists activation in the presence of different concentration of MAPK signaling inhibitors was further characterized by dose-response experiments, further confirming a negative correlation between the amount of IL-22 production and specific signaling inhibitors concentration (Supplementary Fig. 7).

These findings demonstrate that distinct signaling pathways in BMDCs mediate IL-22 and IL-23 release. The data underline differences in MAPK signaling requirements for IL-22 and IL-23 during PRRs ligation, suggesting a critical role of the intracellular pathways in the regulation of these cytokine genes in BMDCs.

### IL-22 is regulated by AP-1/JunD, NFkb, and AhR, but not by NFAT and RORgt

Cytokine genes are usually under the control of multiple transcription factors that coordinately bind to the promoter region to induce the appropriate cytokine response in a cell specific manner^37^,^38^. MAPK signaling transduction pathways use AP-1 as a converging point to regulate gene expression. The transcription factor AP-1 is composed by Jun (c-Jun, JunB, and JunD) and Fos (c-Fos, FosB, Fra1, and Fra2) family members and plays a central role in regulating gene transcription in various biological processes^39^.

**Figure 7:**
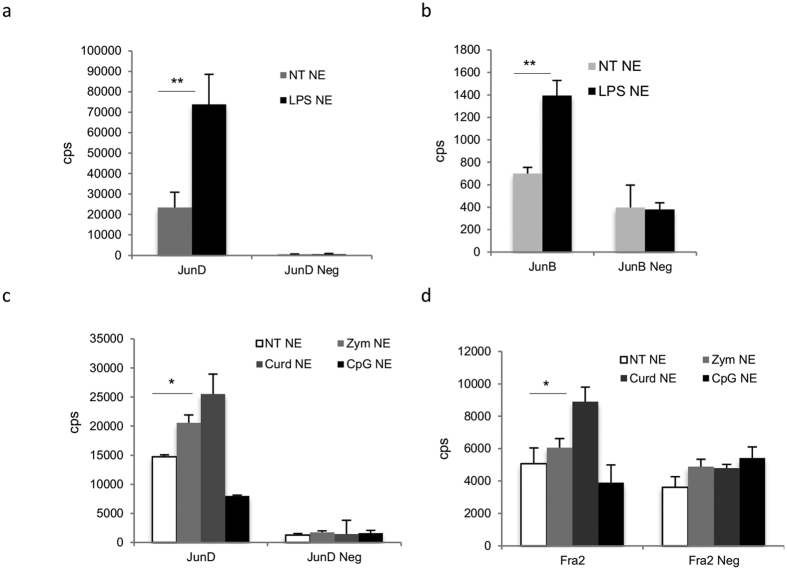
Subunits JunD and JunB translocate into the nucleus during LPS activation. BMDCs were stimulated with LPS (5 ug/ml), CpG (5 ug/ml), zymosan (10 ug/ml), and curdlan (10 ug/ml). After 1 h, nuclear extracts for AP-1 subunit nuclear translocation assay were prepared. (**a**,**b**) Analysis of the JunD and JunB subunits after LPS induction; (**c**,**d**) Analysis of JunD and Fra1 subunits after CpG, curdlan, and zymosan induction. Data represent the mean values of two independent measurements. Student’s T test statistical significance is shown (**p < 0.01; *p < 0.05). Neg, TFA negative probe, Cps, counts per seconds.

To determine the principal AP-1 subunits induced in DCs in response to LPS and therefore relevant for IL-22 gene transcription, nuclear extracts were prepared from BMDCs treated with LPS for 1 hr. The AP-1 members (c-Fos, FosB, Fra1, Fra2, c-Jun, JunB and JunD) were tested by the AP-1 specific subunits via Transcription Factors assay. Only JunD and to a lesser extent, JunB were detected in the cellular nuclear extracts after LPS stimulation in BMDCs (Fig. 7a,b) indicating that the homodimers JunD-JunD or JunD-JunB were the main AP-1 complexes present in the nuclear fractions of BMDCs in response to LPS. Conversely, heterodimers composed of JunD-Fra2 were detected in the nuclear extracts of BMDCs stimulated by zymosan and curdlan, but not by GpG (Fig. 7c,d). The results suggest that different AP-1 subunits control the cellular specific response to PRRs stimulation that will lead to a differential cytokine gene transcription induction.

**Figure 8:**
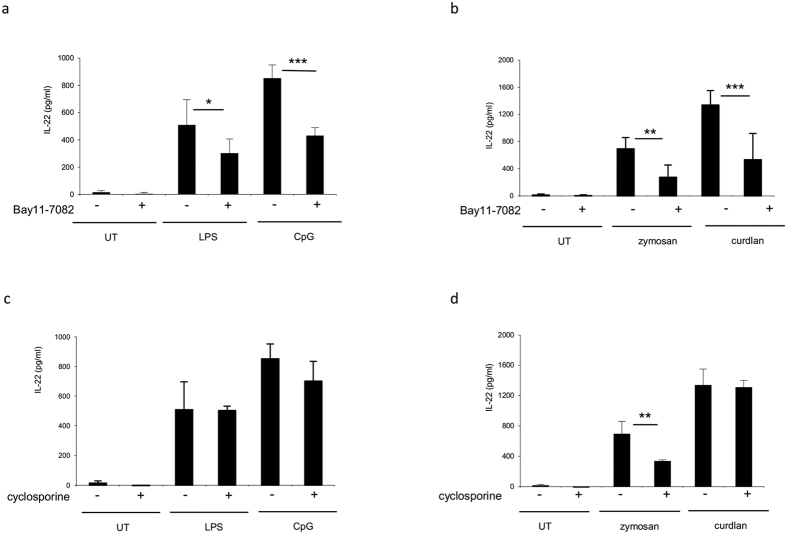
NF-kB, but not NFAT, is involved in IL-22 production in BMDCs. BMDCs (1,5 × 10^6^cells/ml) were pretreated for 30 min with NFkB inhibitor Bay11-7082 (10 uM) or NFAT inhibitor cyclosporine (10 uM). BMDCs were then stimulated with **(a**,**c)** TLR agonist LPS (5 ug/ml) and CpG (5 ug/ml) or **(b**,**d)** zymosan (10 ug/ml) and curdlan (10 ug/ml). After 20 h, supernatants were collected and tested for IL-22 production by ELISA. The data represent mean values of three independent measurements (±SD). Student’s T test statistical significance is shown (***p < 0.001; **p < 0.01; *p < 0.05).

Given that AP-1 is required for IL-22 gene transcription, we asked whether other transcription factors were involved in IL-22 release in BMDCs. NFkb and NFAT regulate pro-inflammatory cytokines in DCs. Therefore we treated BMDCs with IKKa/NFκB inhibitor (BAY117082) and with the calcineurin/NFAT inhibitor (cyclosporine) for 30 min followed by PRR ligands stimulation. Interestingly, a reduction of 40.5% and 49.5% of IL-22 production was detected when cells were stimulated with LPS and CpG, respectively, in the presence of BAY117082 (Fig. 8a and Supplementary Fig. 7c). A much stronger reduction (60.5% and 60.1%) was observed when zymosan and curdlan were used to stimulate the cells (Fig. 8b). Inhibition of NFkB had a greater impact on IL-23 protein release, indicating an exclusive dependence on NFkB for IL-23 gene transcription (Supplementary Fig. 6a,b). We therefore conclude that NFkB activation is necessary, but not sufficient, for IL-22 gene transcription compared to transcription factor AP-1.

Surprisingly, inhibition of NFAT signaling did not affect IL-22 production during PRRs stimulation using LPS, CpG, and curdlan; nevertheless, a cytokine reduction of 52% was observed during zymosan-mediated activation (Fig. 8d). As a control, NFAT inhibitor was tested on IL-2 release induced by CpG, zymosan, and curdlan (Supplementary Fig. 6c). The mechanism underlying the reduction of IL-22 induced by cyclosporine during activation with zymosan is not entirely clear. One possibility is that zymosan engages additional regulators that bind and inhibit NFAT to directly regulate IL-22.

**Figure 9:**
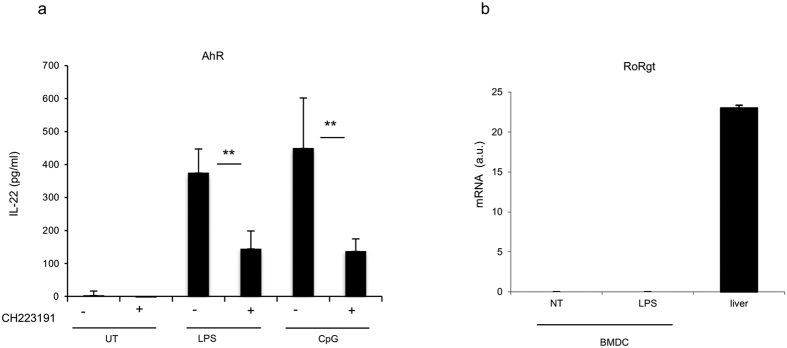
AhR, but not RoRgt, is necessary for IL-22 production in BMDCs. (**a**) Cells (1,5 × 10^6^cells/ml) were pretreated for 30 min with AhR antagonist CH223191 (10 uM). Supernatants were collected and analyzed for IL-22 production 20 h post stimulation with LPS (5 ug/ml) and CpG (5 ug/ml). (**b**) RoRgt is not expressed in BMDCs. BMDCs (1,5 × 10^6^cells), and samples from liver cells were collected in trizol. After reverse transcription, qRT-PCR was performed. QRT-PCR assays were carried out in duplicate. The data represent the mean values of four (**a**) and three (**b**) independent experiments (±SD). Student’s T test statistical significance is shown (**p < 0.01).

Aryl hydrocarbon receptor (AhR) and RoRgt have been reported to be important for IL-22 production in lymphocytes. To investigate the implication of AhR in IL-22 synthesis induced by CpG and LPS, we used the well-described AhR antagonist CH-223191^40^. BMDCs were pretreated with CH-223191 (10 uM) for 30 min and then stimulated for 20 h with LPS and CpG. Results show that IL-22 production by PRR-ligands was dependent on the AhR pathway (Fig. 9a).

To investigate whether RORgt is critically required for IL-22 production induced by LPS, we first tested RORgt expression in BMDCs before and after LPS treatment. The level of RORgt was measured by qRT-PCR. Gene expression analysis revealed that RORgt is not expressed in resting or in LPS-stimulated BMDCs as compared to liver cells (Fig. 9b).

Therefore, we can conclude that RORgt is not required for IL-22 production in BMDCs as opposed to the requirement of the nuclear receptor for Th17 cells^41^. Our data suggest that a specific intracellular signaling cascade, able to specifically regulate IL-22 gene transcription, exists in BMDCs.

### Splenic CD11c+ cells release IL-22 in response to LPS *in vivo*

**Figure 10:**
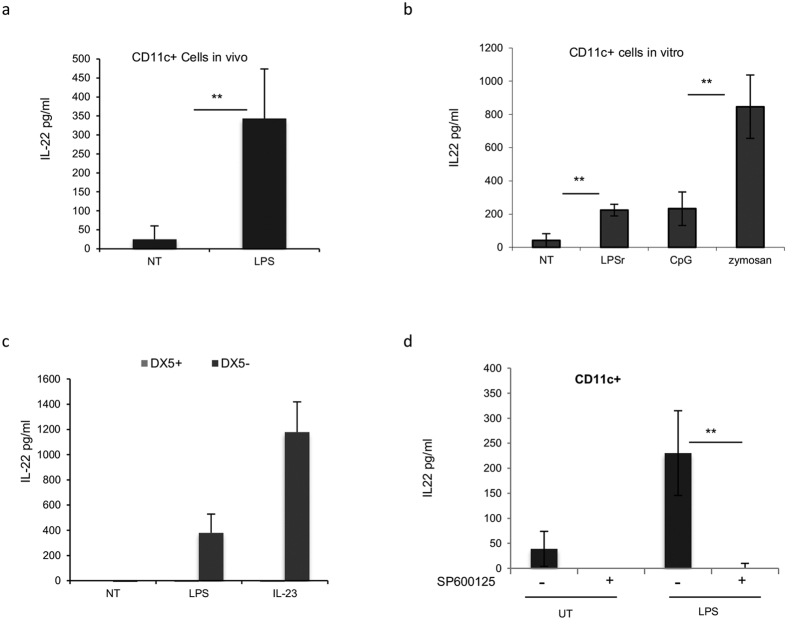
CD11c+ cells release IL-22 both *in vitro* and *in vivo* after stimulation with LPS. (**a**) A group of mice (N = 5) were treated i.v. with 50 ug of LPS. After 3 h, the spleens were removed and CD11c+ cells were purified by magnetic beads isolation and cultured for 20 h. IL-22 was measured by ELISA. (**b**) *Ex vivo* CD11c+ cells were purified from the spleens of a group of mice (N = 4). CD11c+ cells were then stimulated with LPSr (5 ug/ml), CpG (5 ug/ml), and zymosan (10 ug/ml). After 20 h, supernatants were collected and tested for IL-22 production by ELISA. (**c**) DX5+ cells from RAG2 KO mice (N = 3) were purified by magnetic beads isolation. DX5+ and DX5- negative fraction were *in vitro* stimulated with LPS (5 ug/ml) and rmIL23. After 20 h, supernatants were collected and tested for IL-22 production by ELISA. (**d**) CD11c+ cells were pretreated for 30 min with JNK inhibitor SP600125 (20 uM) and then stimulated with LPS (5 ug/ml). After 20 h, supernatants were collected and tested for IL-22 production by ELISA. The data represent the mean values of at least three independent experiments (±SD). Student’s T test statistical significance is shown (**p < 0.01).

To verify that *in vivo* DCs are also able to secrete IL-22 in response to LPS, we performed a set of *in vivo* experiments. A group of five C57BL/6 mice were treated i.v. with LPS (50 ug/mouse) or with PBS. After 3 h, splenic CD11c+ DCs were isolated and cultured for 20 h. IL-22 protein levels were measured by ELISA. As shown in Fig. 10a, CD11c+ cells isolated from the spleen of LPS-treated mice were able to secrete IL-22.

To determine whether *ex vivo* CD11c+ cells stimulated *in vitro* with LPS were able to produce IL-22 protein, we treated splenic CD11c+ cells with 10 ug/ml LPS for 20 h and then determined IL-22 with ELISA. The data once again confirmed that CD11c+ cells release IL-22 in response to LPS, CpG and zymosan (Fig. 10b). Finally, to exclude the responsibility of other CD11c+ populations for IL-22 induction in the spleen, we purified DX5+ NK cells from Balbc/RAG2 KO mice. The cells were stimulated with either IL-23 or LPS, and cytokine concentration was measured 20 h after stimulation. As shown in Fig. 10c, DX5+ NK cells were not able to secrete IL-22 upon LPS and IL-23 stimulation, confirming that a splenic population expressing CD11c+ other than NK cells is able to release IL-22 upon TLR4 stimulation. The negative fraction of this cell preparation (DX5−) was used as a positive control. To verify that JNK was involved in IL-22 release also by *ex vivo* cells, we pretreated CD11c+ cells for 30 min with SP600125 (targeting c-Jun N-terminal kinases) and subsequently cultured with LPS (5 μg/ml). The production of IL-22 was determined by ELISA after 20 hr (Fig. 10d). As shown in Fig. 10d, the expression of IL-22, mediated by LPS is controlled by JNK kinases also in CD11c+ cells.

Finally, attempts to verify whether IL-22 expression in *ex vivo* splenic DCs is independent from IL-23, as it was the case for BMDCs, were not conclusive, as *ex vivo* CD11c+ cells were able to secrete IL-22 in the presence of IL-23 (data not shown). Purification of cells by either extensive FACS or beads sorting technologies were hardly generating homogenous populations that may have biased the *ex vivo* analysis. Therefore, we are unable to conclude that the IL-23-independent intracellular pathway discovered in BMDCs is also operating in *ex vivo* splenic CD11c+ cells.

## Discussion

Myeloid antigen-presenting cells (APCs) tailor immune responses to the pathogen involved through the production of specific pro- and anti-inflammatory cytokines. It is becoming increasingly clear that the ultimate cytokine profile produced by myeloid APCs crucially depends on the interaction between multiple pathogen recognizing receptors. DCs are activated by different signals, such as microorganism products, pro-inflammatory cytokines, and other molecules. Several studies show that PRR receptor ligation induces the expression of a variety of molecules related to function, maturation, and induction of adaptive immunity by DCs. Specifically, DCs through production of PRR-induced cytokines participate in determining the type of generated effector responses. Therefore, it is important to define the molecular pathways regulated in DCs in response to PRRs signals.

Gene expression analysis at the host-pathogen interface has shed light on the molecular mechanisms induced in DCs in response to microbial interactions^1^,^7^,^42^. In addition, genetic profiles obtained by using whole live microbial organisms have helped to define the molecular pathways induced during multiple PRR regulation, generating a general overview of the inflammatory response induced in DCs^43^. Moreover, a dissection of the single PRR contribution to the release of a specific cytokine profile has been extensively demonstrated both *in vitro* and *in vivo*, with the best characterized of which is TLR4 and its ligand LPS^44^,^45^.

In this study, we describe a novel pathway induced during PRR stimulation in BMDCs, leading to the secretion of the IL-22 cytokine.

Recently, there has been an increased interest in understanding the role of IL-22 in health and disease with the aim of exploiting this specific cytokine in therapy. IL-22 is released by leukocytes but specifically targets non-hematopoietic cells, thus providing a connection between immune system cells and parenchymal cells. The IL-22 cytokine regulates other molecules involved in tissue inflammation, immunosurveillance, repair, and homeostasis^12^,^13^,^17^,^18^,^46^,^47^. Dysregulation of IL-22 expression can occur in human diseases and in preclinical models, e.g. the crucial role of IL-22 in regulating homeostasis of epithelial cells at barrier surfaces has been identified in a mouse model infection.

IL-22 can be induced by antigen-experienced T cells, such as Th17 or Th22, and by innate lymphocytes, such as NK cells, γ/δ T cells, and lymphoid tissue inducer cells^12^,^24^,^48^,^49^,^50^. Detailed analysis of IL-22 gene regulation shows the requirements of the cytokines IL-12, IL18, IL-21, and IL-6 for cytokine induction in Th1 cells and Th17 cells^12^,^35^,^51^.

Overall, IL-23 is essential in promoting IL-22 production in the mentioned cell types. Indeed, IL-23 alone is able to induce IL-22 production from CD4+ and CD8+ T cells, γ/δ T cells, monocytes, and LTi cells^25^,^35^,^52^. *In vivo*, IL-22 induction is significantly compromised in IL-23 deficient mice under various immune challenges^19^,^53^,^54^,^55^.

Transcription factor (TF) requirements for IL-22 release in T cells have been extensively studied. RORγt and RORα are essential downstream regulators of IL-6 and IL-23 in T cells^56^,^57^,^58^. Notch and STAT3 signaling all are involved in IL-22 gene regulation in T cells^59^.

Although several TFs regulate the expression of cytokine genes, TF combination controls specific sets of cytokines. Therefore, understanding how cytokine genes are fine-tuned by specific TFs can lead directly to their control during disease.

The above mentioned studies primarily addressed the response of T cells in different mouse model settings. Few data exist on the detailed regulation of the IL-22 in DCs, although some studies report that monocytes and DCs produce no or very low levels of IL-22 upon IL-23 stimulation^60^,^61^.

The data we present here confirm that BMDCs are not able to release IL-22 after IL-23 stimulation. We report the discovery of a novel pathway operating specifically in BMDCs. This pathway is induced by PRRs stimulation, directly leading to IL-22 induction in BMDCs in a Myd88-dependent, but IL23-independent mechanism. In addition, we characterized the intracellular signaling pathways controlling IL-22 gene transcription in BMDCs and show that cytokine release requires activation of MEK1 ERK/JNK phosphorylation. The transcription factor complex AP-1 is the final target of MAP kinase signaling pathways^62^. AP-1 complexes are composed of members of the Jun and Fos families and bind to specific control elements present in the promoters of genes regulating cell differentiation and proliferation^62^,^63^. The Jun proteins can both homo- and heterodimerize with Fos members to form transcriptionally active complexes. We found that in response to LPS, the AP-1 complexes that were nuclear translocated were mainly formed by the subunits JunB and JunD, leading to the conclusion that AP-1 complex JunD/JunB dimers is required for IL-22 transcription in conjunction with other transcription factors. It is tempting to speculate that the type of AP-1 subunits induced may determine the different cytokines finally released by the cells in response to different PRR ligands. IL-22 promoter analysis confirmed that multiple AP-1 transcription binding sites together with GATA, STAT, AHR, and CREB binding sites are present in the promoter region of the IL-22 gene (data not shown).

The TFs confirmed in our analysis were NFkB and AhR, but not RORγt or NFAT for IL-22 gene regulation in BMDCs (Figs 7 and 8). In contrast to T cells, we could not confirm the involvement of RORγt control in IL-22 production in BMDCs. We therefore conclude that IL-22 cytokine release in BMDCs is under a RORγt-independent mechanism. These data further suggest that it is important to determine the intracellular signaling cascade in the specific tissue and/or cell type in order to correctly manipulate the cytokine response in health and disease.

In addition, although macrophages express PRR receptors, we were not able to measure IL-22 in this cell type, suggesting that BMDCs possess a unique molecular machinery that renders the cells suitable to respond to pathogens with additional molecular mechanisms operating in a cell specific manner. Therefore, we speculate that BMDCs are equipped with molecular signals (intracellular or membrane-bound) making them suitable to respond to pathogens not only to regulate the nascent immune system cells, but also to directly regulate the cross talks with parenchymal cells. This feature makes BMDCs a unique cell type at the host-pathogen interface with a clear distinct functional capacity compared to macrophages.

In conclusion, our findings have established that BMDCs are an early source of IL-22 and able to secrete the cytokine in response to PRR ligands independent of IL-23. The exact identification of the specific intracellular pathways operating in BMDCs will enable the development of specific target strategies aiming at reinforcing this functional activity. Collectively, our data document a network of interactions affecting IL-22 level and outcome from PRR activation in a cell-specific manner.

## Methods

### Ethics statement

All animal experiments were performed using protocols approved by the University of Milano-Bicocca Animal Care and Use Committee. All experimental procedures were carried out in strict accordance with the 2003/65/CEE European directive for animal experimentation. Protocols used in this study were approved by the Italian Ministry of health under the protocol number 3–2001.

### Cell cultures

D1 cells were maintained *in vitro* in Iscove’s modified Dulbecco’s medium (IMDM, Euroclone) supplemented with 10% heat-inactivated fetal bovine serum (Gibco, origin: Australia), 100 IU/ml penicillin, 100 μg/ml streptomycin, 2 mM L-glutamine (all from Euroclone) and 50 μM β-mercaptoethanol (Sigma) plus 30% R1 medium (supernatant from NIH3T3 fibroblasts transfected with GM-CSF). Cells were incubated at 37 °C under 5% CO_2_.

Bone marrow-derived Dendritic Cells (BMDC) from C57BL/6 wild-type (WT) and MyD88 knock-out (KO) mice were cultured in IMDM (Euroclone, Milan, Italy) supplemented with 10% heat-inactivated FBS, 2 mM L-glutamine, 100 U/ml penicillin, 100 μg/ml streptomycin (all from Euroclone), 50 μM β-mercaptoethnol (Sigma), (IMDM complete medium), and 20% supernatant of GM-CSF transduced B16 tumor cells (20 ng/ml GM-CSF). Isolated cells were seeded at 6 × 10^6^ cells/petri dish with 10 ml of grow medium (IMDM complete, 20% B16). Cells were incubated at 37 °C under 7% CO_2_. At day 3, 10 ml of fresh medium were added; at day 6, 10 ml of medium were changed. After 7–10 cultivation days, cells were analyzed by Flow cytometry (FACS) for CD11c expression and used in assays when 75–85% of the cells were CD11c-positive.

Bone marrow-derived macrophages (BMDM) from C57BL/6 wild-type (WT) were generated, as previously described, by culturing cells in medium containing M-CSF^37^.

### *Ex-vivo* cells

Primary splenocytes were isolated by breaking up the spleen of WT or Myd88−/− C57BL/6 mice. For some experiments BALB/c mice were used. The obtained cells were cultured (2 × 10^6^ cells/ml) in complete IMDM in an incubator under 5% CO_2_ at 37 °C or were processed, after red blood cell lysis, to purify different cell populations. In the first protocol, the splenocytes were stained with anti-CD11c magnetic beads (Miltenyi Biotech) or with biotin anti-CD49b (Biolegend, clone DX5) and magnetically labeled with streptavidin beads (Miltenyi Biotech). Cells were positively selected with MS columns, according to the manufacturer’s recommendations. DCs obtained were 85% CD11c-positive and 95% MHCII-positive. NK cells obtained were 97% DX5-positive. In the second protocol, the splenocytes were depleted of T cells (CD3+, TCR+), B cells (Cd19+), NK cells (DX5+), and granulocytes (Gr1+). The remaining cells were then stained with anti-CD11c and sorted on a Beckman Coulter MoFlo. DCs obtained (97% CD11c positive) were cultured at 2,5 × 106 cells/ml in complete IMDM. Alternatively, the splenocytes were stained with anti-CD4, anti-CD8, and anti-CD19 and sorted on a Beckman Coulter MoFlo. The purity was higher than 97% for all cell populations. All antibodies used were from Biolegend, San Diego, CA, USA. Purified cells were cultured (2,5 × 10^6^ cells/ml) in complete IMDM in an incubator under 5% CO_2_ at 37 °C.

### Cell stimulation and cytokine detection

TLR ligands set III (Apotech, Axxora) were used to stimulate BMDCs. Mouse recombinants IL-23 (eBioscience or R&D), IL-18 (MBL), and IL-15 (eBioscience) were used to stimulate spleen-derived cells. IL-22 protein levels were measured by ELISA (Bender MedSystems) according to the manufacturer’s instructions.

### *In vivo* experiments

C57BL/6 WT mice were purchased from Charles River and maintained in our animal facility at the University of Milano-Bicocca, Milan, Italy. C57BL/6 Myd88−/− were kindly provided by S. Akira. All experiments were performed using protocols approved by the University of the Milano-Bicocca Animal Care and Use Committee. Mice were housed under pathogen-free conditions and maintained on a regular 12: 12 hour light: dark cycle with food and water ad libitum.

Seven-week-old C57BL/6 mice were injected with PBS (control) and rLPS (50 mg/mouse, Alexis, serotype R515) or infected with different bacteria (*Listeria monocytogenes*, *Listeria innocua*). Spleens were removed, at different time points, and placed in RNA-later solution (Ambion) to preserve mRNA. In other experiments, the spleen was isolated at 3 hours post injection and used for the isolation of CD11c+ cells.

### Bacteria

*Lactobacillus paracasei* (Lp) B21060 strain was kindly supplied by Bracco SpA (Milano). This strain was grown in MRS Broth (Fluka) at 37 °C under agitation, to an OD600 of 0.8, corresponding to the exponential growth phase. *Lactococcus lactis* (Lc) MG1363 strain was kindly provided by Jerry Wells (TNO, Netherlands). This bacterium was grown in M-17 broth, supplemented with 0,5% glucose, at 30 °C in a water bath to an OD600 of 1, corresponding to the log growth phase. *Listeria innocua* (Li) was grown in brain heart infusion (BHI, Fluka) at 37 °C under agitation to an OD600 of 0.6, corresponding to the exponential growth phase. *Listeria monocytogenes* (Lm) EGD pNF8 was kindly provided by Pascal Cossard and Olivier Dussurget from Institut Pasteur, Paris, France. This strain carries the pNF8 plasmid, which contains the gene encoding the GFP protein. The replicative plasmid was maintained in the strains by growing them on brain heart infusion (BHI, Fluka) containing 5 μg/ml of erythromycin. *L. monocytogenes* was grown to a mid-logarithmic phase (OD600 = 0.6). Bacteria were stored as 10% glycerol stocks at –80 °C in small aliquots until use. The concentrations were quantified by plating serial dilutions on agar plates and counting colonies after growth at 30–37 °C for 24–36 hours.

For *in vitro* infections, bacteria were thawed from glycerol stocks, washed in PBS, diluted in appropriate media and added onto cells at the selected multiplicity of infection (MOI). At 1 h post infection, bacteria were removed and fresh medium containing gentamycin (50 μg/ml) was added. The real number of bacteria was verified by plating serial dilutions of the diluted stimulus on appropriate agar plates.

### Microarray experiments and data analysis

D1 cells were infected with different Gram+ bacteria: *L. paracasei* (MOI 1:1000), *L. lactis* (MOI 1:1000), *L. monocytogenes* (MOI 1:40), and *L. innocua* (MOI 1:1000). We harvested 107 D1 cells in the immature state or after 4 h, 8 h, or 24 h of stimulation. Total RNA was isolated with trizol reagent (invitrogen) and purified on a Qiagen RNeasy column (Qiagen) to remove small fragments. Samples were processed as previously described and hybridized onto the MOE430A GeneChip (Affymetrix) using the recommended procedures. A single log scale normalized expression measure for each probe set was obtained from the low-level data files (CEL files) by the robust multiarray analysis (RMA) procedure. The data were subjected to Z-score-based transformation. For the selection of differentially expressed gene (DEG), we used the AMDA (Automated Microarray Data Analysis) software based on LIMMA (Linear Models for Microarray Data).

### Quantitative Real Time Polymerase Chain Reaction (q-Real Time PCR)

Total RNA was isolated with trizol reagent (invitrogen) and purified on a Qiagen RNeasy column (Mini or micro kit, Qiagen). We followed the manufacturer’s recommendations for cells or for tissues. When processing tissues, disruption and homogenization step was performed using the TissueLyser (Qiagen). DNase digestion was carried out in the column during RNA extraction (RNase-free DNase Set, Qiagen). RNA quantity and quality were evaluated spectrophotometrically (NanoDrop ND-1000 Spectrophotometer, Thermo Scientific). Generally, we reverse transcribed 1 μg of total RNA with random primers (High Capacity cDNA Reverse Transcription Kit, Applied Biosystems). Quantitative Real Time PCR (qRT-PCR) was performed on 10 ng of total cDNA using primer sets specific for the selected genes and the 18 s or PPIA housekeeping genes. All qRT-PCR assays were run on an ABI 7500 machine with Power SYBR Green PCR Master Mix (Applied Biosystems); each measurement was performed in duplicate. Relative quantification was performed using the comparative threshold cycle (Ct) method, and results were expressed in arbitrary units (AU). Expression levels were calculated as 2^−ΔCt^, whereas fold changes were calculated using the 2^−ΔΔCt^ equation.

### FACS analysis

To assess the maturation of BMDCs and to verify the quality of purification processes, flow cytometry analysis was performed. In order to block non-specific binding of antibodies to the FcγIII and FcγII receptors, we used the 2.4G2 antibody. We used the following antibodies: anti-CD11c-APC, anti-CD86-PE, anti-MHCII-PE, anti-NK1.1-PE, anti-CD4-APC, anti-CD8-FITC, anti-B220-FITC. The corresponding primary-labelled isotype control antibodies were used for staining controls. Cells were analyzed on a FACScalibur (BD, Heidelberg, Germany) using the corresponding CellQuest software.

### AP-1 subunits TF Assay

BMDCs were stimulated with LPS for 1 hr and nuclear extracts were prepared using the Nuclear Extraction kit (Chemicon). The AP-1 Transcritpion Factor subunits were analysed by using the AP-1 family EZ-TFA Transcription Factor Assay Chemioluminescent AP-1 Family (Millipore).

### Statistical analysis

Data were analyzed using the Student’s t-test (two-tailed distribution, two-sample equal variance) to determine statistical significance. P values < 0.05 were considered statistically significant.

## Additional Information

**How to cite this article**: Fumagalli, S. *et al.* IL-22 is rapidly induced by Pathogen Recognition Receptors Stimulation in Bone-Marrow-derived Dendritic Cells in the Absence of IL-23. *Sci. Rep.* **6**, 33900; doi: 10.1038/srep33900 (2016).

## Supplementary Material

Supplementary Information
