# Supplementary material for: IL-22 is rapidly induced by Pathogen Recognition Receptors Stimulation in Bone-Marrow-derived Dendritic Cells in the Absence of IL-23
Source: Sci Rep. 2016 Sep 22;6:33900. doi: 10.1038/srep33900 (PMC5031995; doi:10.1038/srep33900)
Supplement: Supplementary Information [file srep33900-s1.doc]

**IL-22 is rapidly induced by Pathogen Recognition Receptors Stimulation in Bone Marrow-derived Dendritic Cells in the Absence of IL-23**

Silvia Fumagalli^1+^, Anna Torri^1+^, Angela Papagna^1^, Stefania Citterio^2^, Federica Mainoldi^1^, and Maria Foti^1, *^

^1^School of Medicine and Surgery, University of Milano-Bicocca, Milan, 20126, Italy

^2^ Department of Biotechnology and Bioscience, University of Milano-Bicocca, Milan, 20126, Italy

*Corresponding author: Email: maria.foti@unimib.it; Phone: (+39) 02-6448 3520 or ext 3530; Fax: (+39) 02-64483552

**Supplementary Figures**

Supplementary Fig. S1


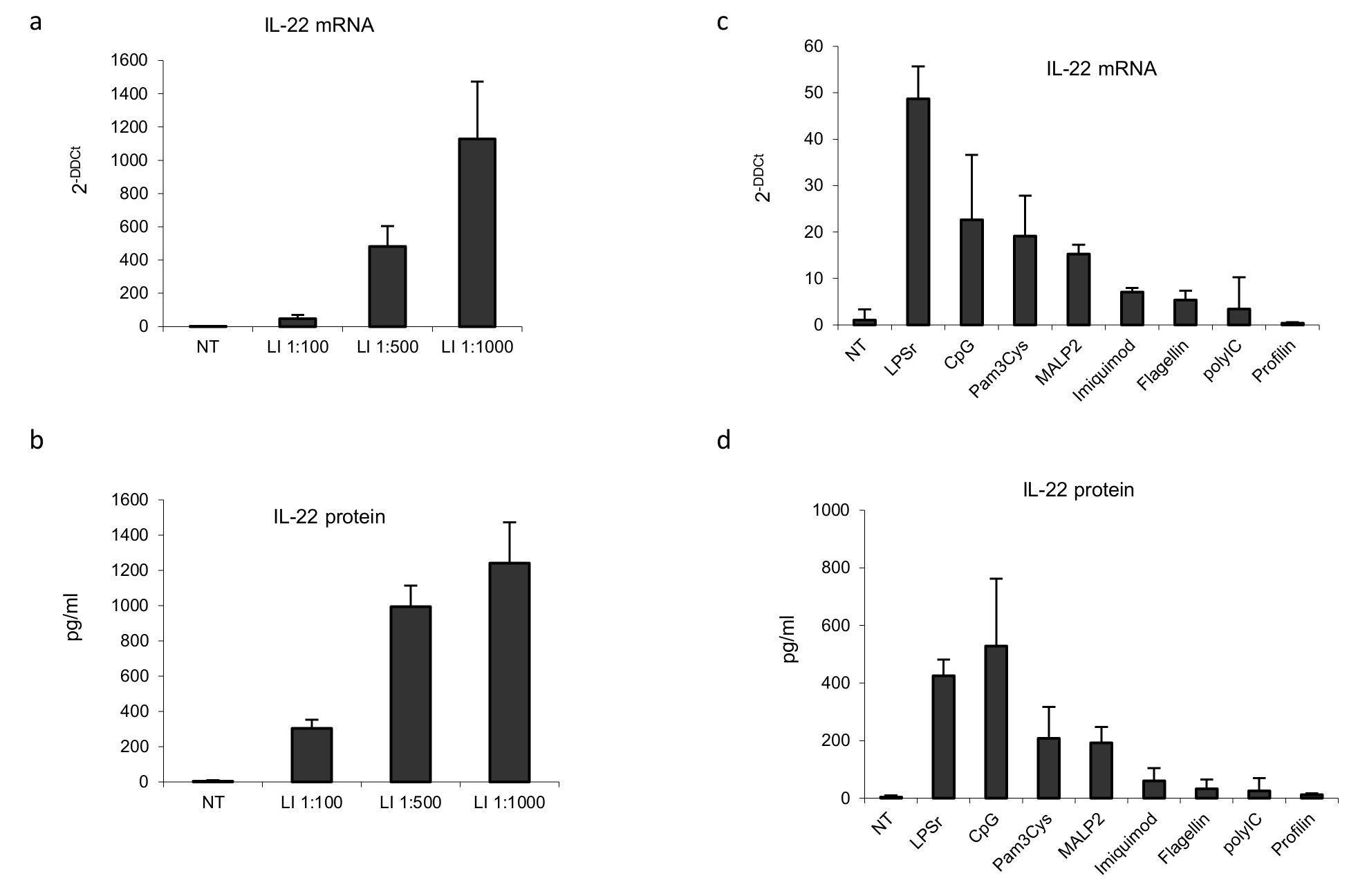


**Supplementary Figure S1:** Detection of IL22 mRNA and protein in BMDCs. (**a-c**) BMDCs (1,5x10^6^ cells/ml) were stimulated with different MOI of the bacteria Listeria innocua (LI) at different multiplicity of infection (MOI 1:100, MOI 1:500, MOI 1:1000) and with TLRs ligands. After 4h, totRNA was extracted and IL22 mRNA measured by QRT-PCR. (**b-d**) Supernatants were collected at time point 20h and tested for IL-22 production by ELISA. mRNA data are normalized against 18s and then expressed as fold stimulation over control (NT, not treated cells). The data represent the mean of two independent experiments (± SD).

Supplementary Fig. S2


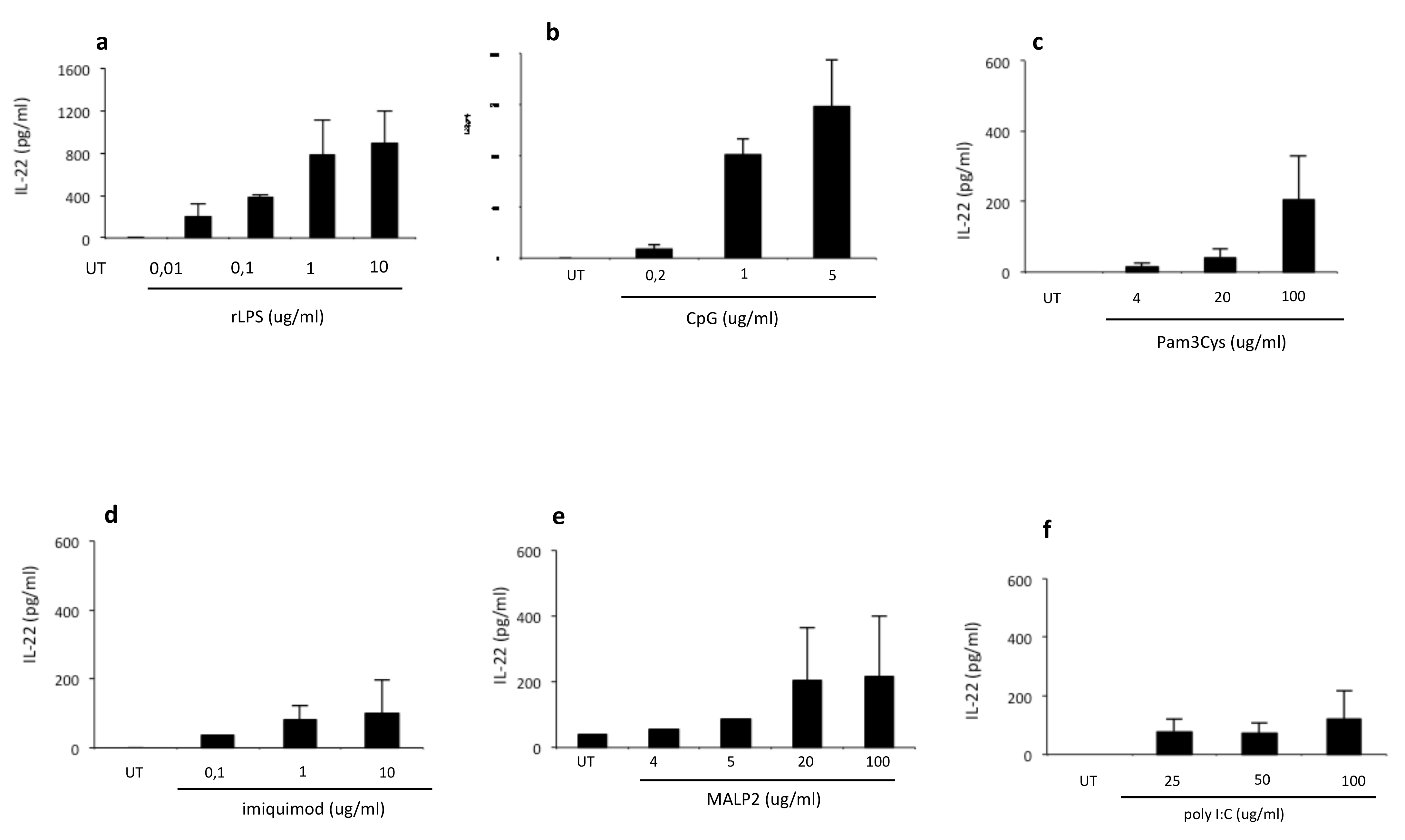


**Supplementary Figure S2:** IL-22 production in BMDCs is dose dependent. (**a-f**) BMDCs (1,5x10^6^ cells/ml) were stimulated with different TLR agonists in dose-response experiments. After 20h, supernatants were collected and tested for IL-22 production by ELISA. The data represent mean values of three independent measurements (± SD).

Supplementary Fig. S3


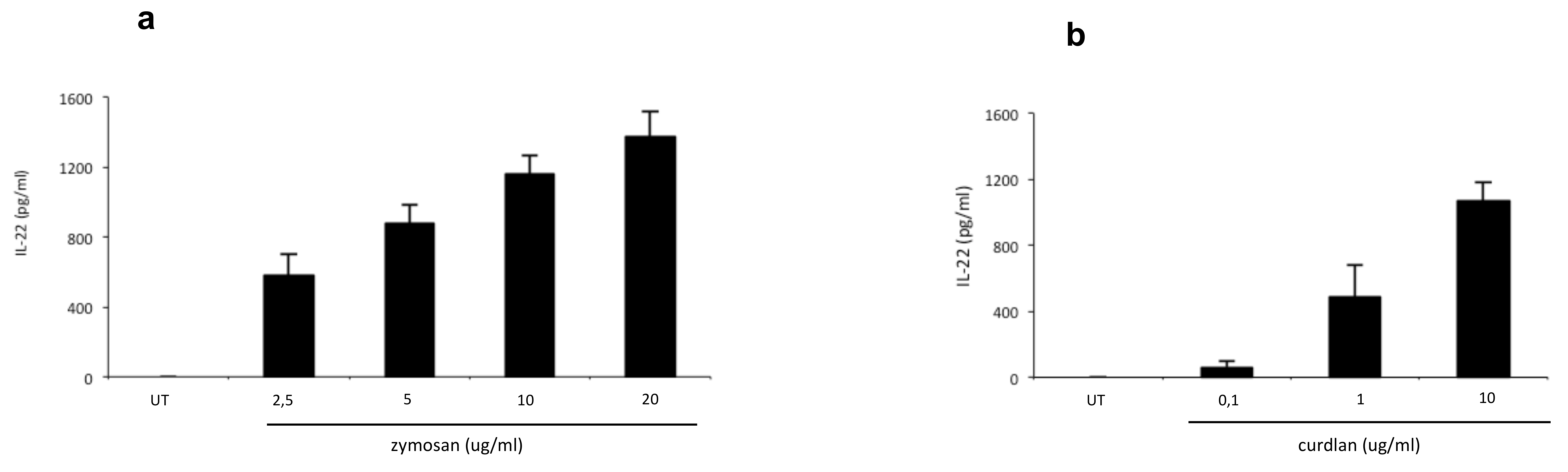


**Supplementary Figure S3:** IL-22 production in BMDCs is dose dependent. (**a-b**) BMDCs (1,5x10^6^ cells/ml) were stimulated with different dectin-1 agonists in dose-response experiments as shown in the figure. After 20h, supernatants were collected and tested for IL-22 production by ELISA. The data represent mean values of three independent measurements (± SD).

Supplementary Fig. S4


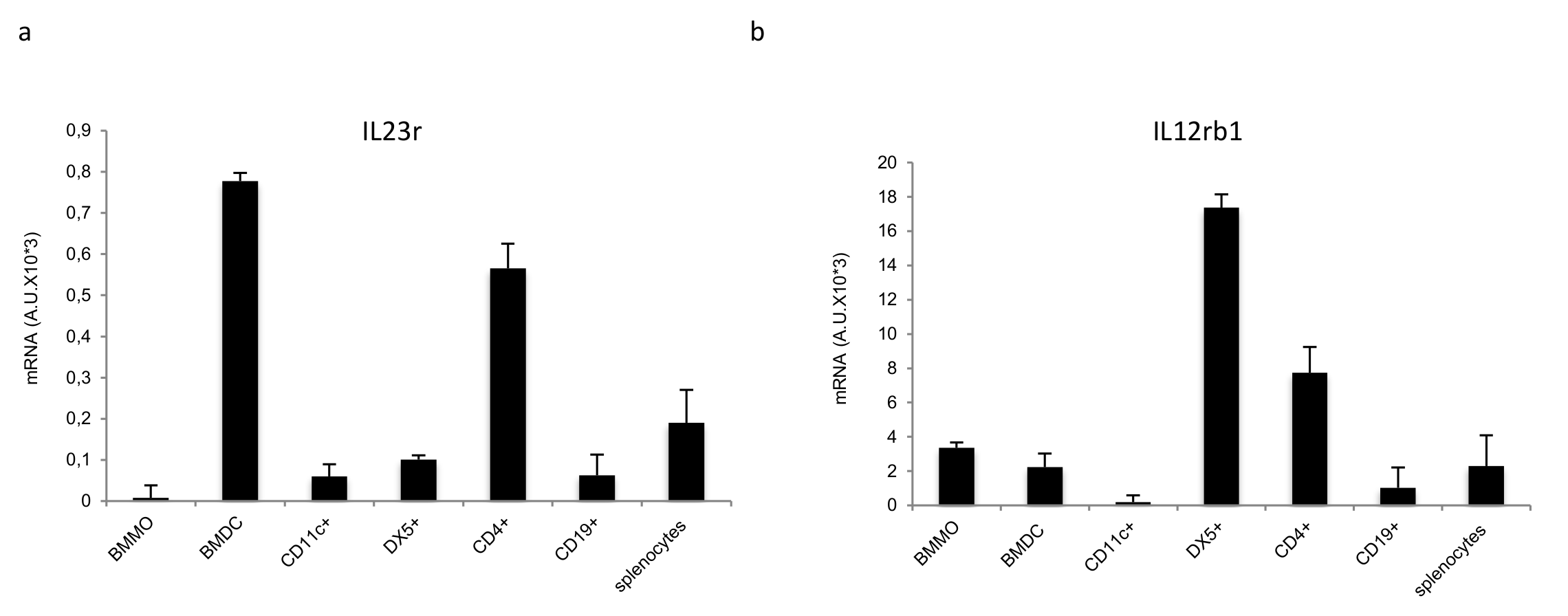


**Supplementary Figure S4**: IL23R subunits (IL23r and IL12rb1) expression in leukocytes. BMDCs, BMDM, ex vivo CD11c+, DX5+, lymphocytes (CD4+, CD19+) and splenocytes were tested by qRT-PCR for (**a**) IL23r and (**b**) IL12rb1subunits. Samples were normalized with PPIA gene expression levels and expressed as arbitrary units. Each measurement was performed in duplicates. The data represent the mean values of two independent experiments (± SD).

Supplementary Fig. S5


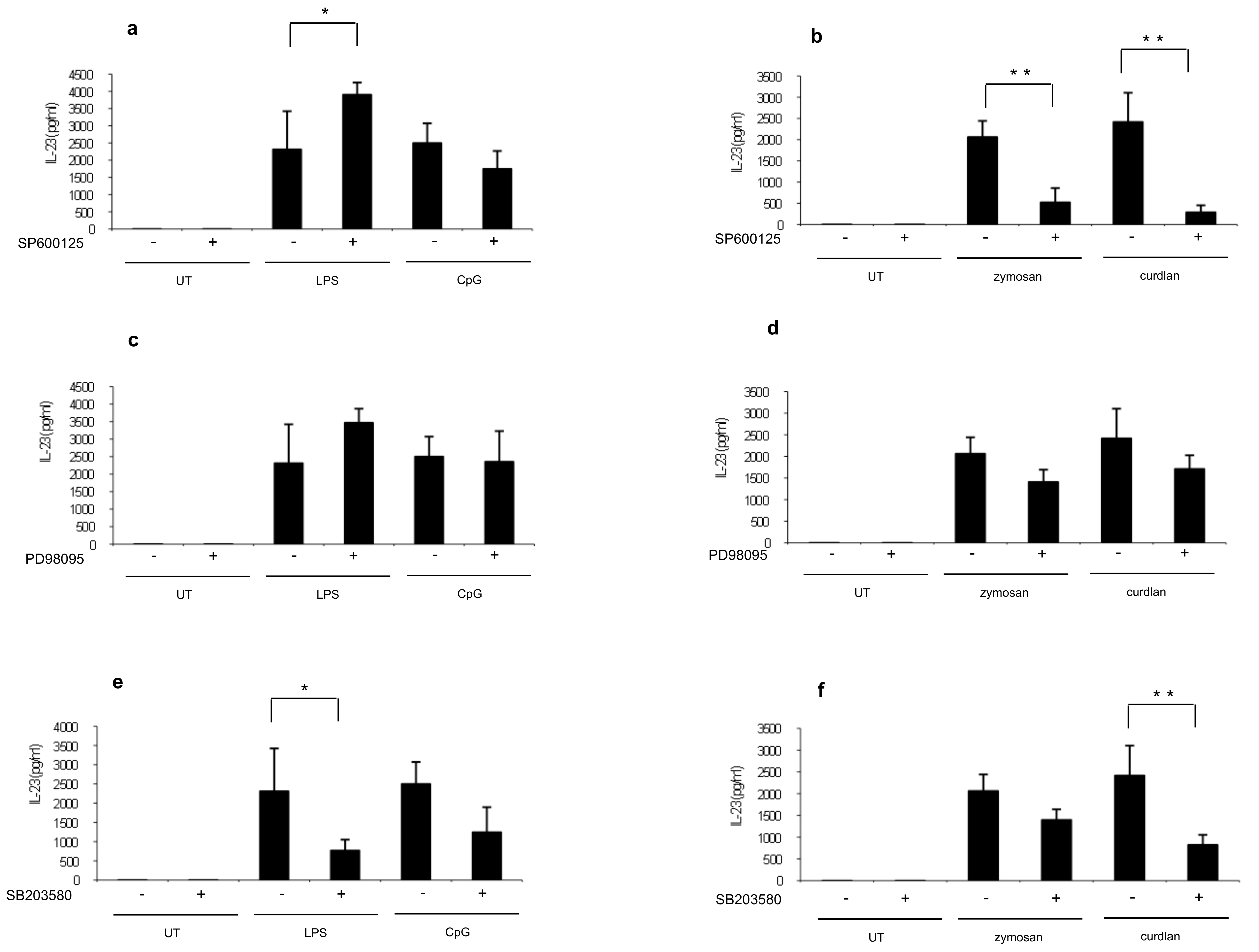


**Supplementary Figure S5:** Role of MAP Kinase in regulating IL-23 production. BMDCs (1,5x10^6^cells/ml) were pretreated for 30 min with (**a**, **b**) JNK inhibitor SP600125 (20uM), (**c**, **d**) ERK inhibitor PD98059 (20uM), and (E, F) p38 inhibitor SB203580 (20uM). BMDCs were then stimulated with (**a**, **c**, **e**) TLR agonist LPS (5ug/ml), CpG (5ug/ml,), and zymosan (10ug/ml) or (**b**, **d**, **f**) curdlan (10ug/ml). After 20h, supernatants were collected and tested for IL-23 production by ELISA. The data represent mean values of three independent measurements (± SD). Student’s T test statistical significance is shown *** p<0,001; ** p<0,01; *p<0,05.

Supplementary Fig. S6


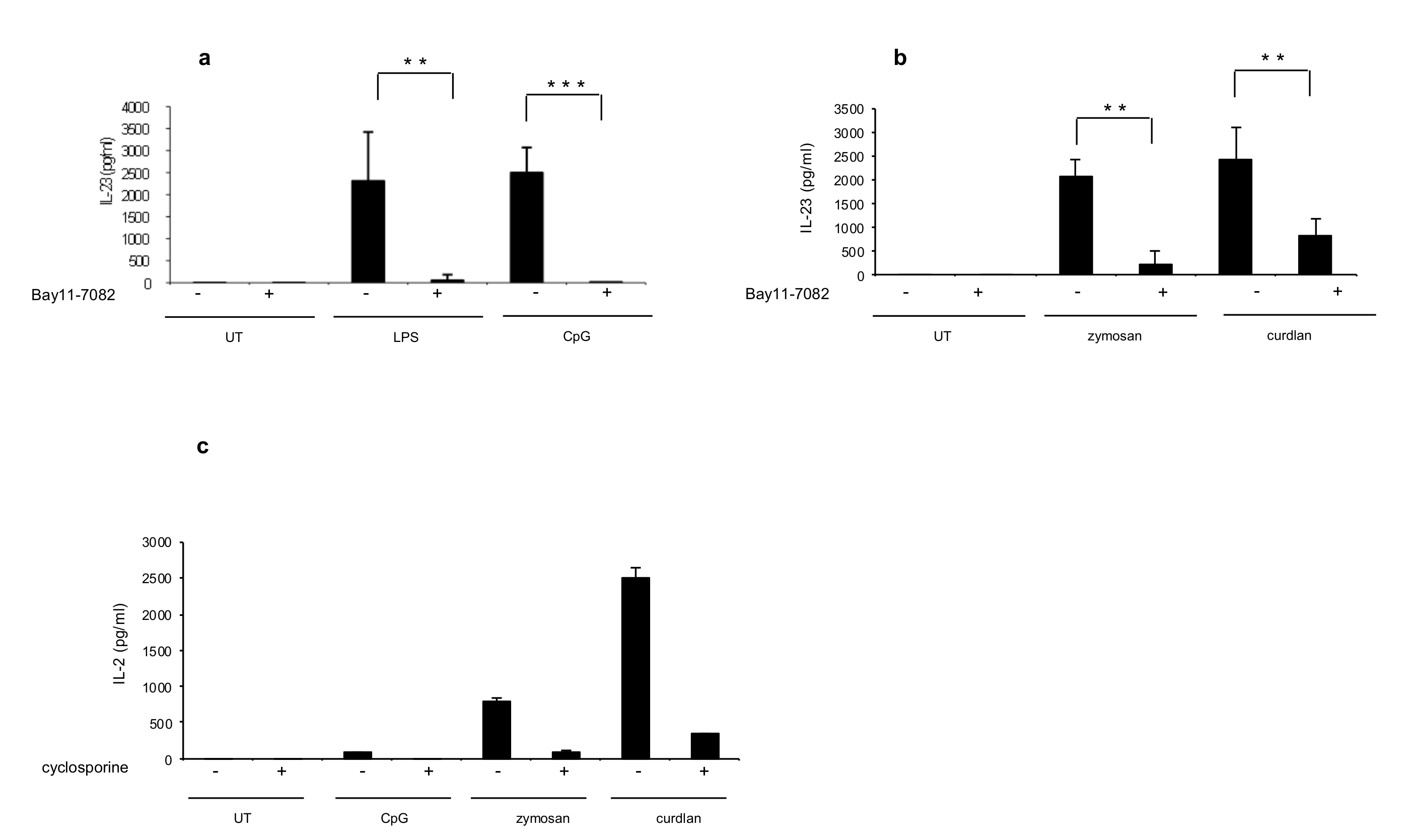


**Supplementary Figure S6:** NFkB and NFAT mediate IL-23 and IL-2 induction respectively. BMDCs (1,5x10^6^cells/ml) were pretreated for 30 min with NFkB inhibitor Bay11-7082 (10uM) or NFAT inhibitor cyclosporine (10uM). BMDCs were then stimulated with **(a)** TLR agonist LPS (5ug/ml) and CpG (5ug/ml) and **(b)** zymosan (10ug/ml) and curdlan (10ug/ml). After 20h, supernatants were collected and tested for IL-23 or IL-2 (**c**) production by ELISA. The data show the mean values of three independent experiments (± SD). Student’s T test statistical significance is shown ( *** p<0,001; ** p<0,01; *p<0,05).

Supplementary Fig.S7


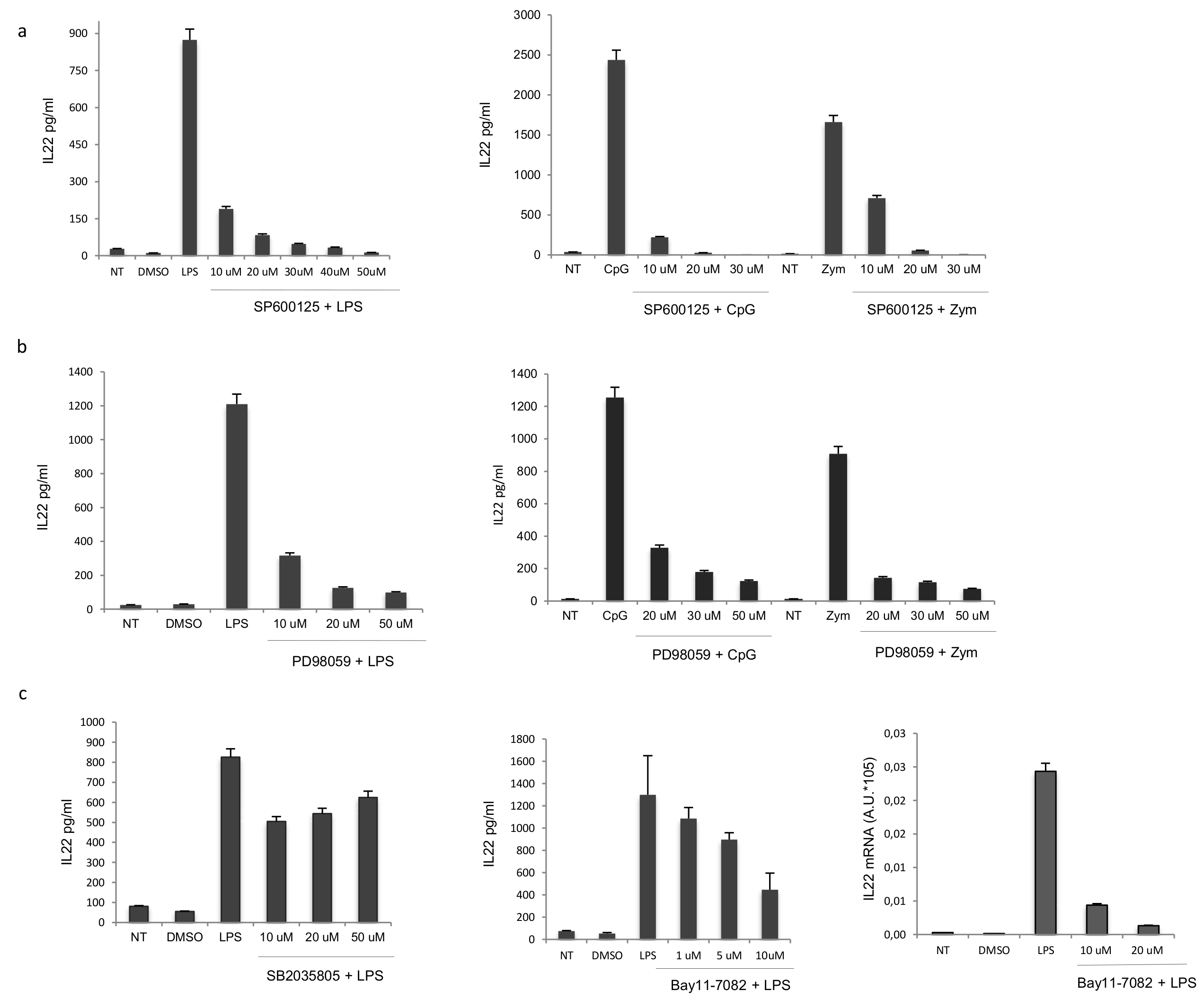


**Supplementary Figure S7: Dose-response analysis of inhibitors on IL22 signaling.** BMDCs (1,5x10^6^cells/ml) were pretreated for 30 min with different concentrations of (**a**) JNK inhibitor SP600125, (**b**) ERK inhibitor PD98059, and (**c**) p38 inhibitor SB203580 and NFKb inhibitor Bay11-7082. BMDCs were then stimulated with TLR agonist LPS (5ug/ml), CpG (5ug/ml,), and zymosan (10ug/ml). After 20h, supernatants were collected and tested for IL-22 production by ELISA. The data represent mean values of three independent measurements (± SD) for (**a**) and two independent experiments for (**b**) and (**c**).
